# Integrating Longitudinal Cohorts for Population Health Research: Mainland and Island-based Puerto Rican Diabetes Study

**DOI:** 10.64898/2026.10.08.26365065

**Published:** 2026-10-11

**Authors:** Andreia Martinho, Martha Tamez, Josiemer Mattei, Elizabeth Petit, Sabrina E. Noel, Bayan Tuffaha, Katherine L. Tucker, Jose M. Ordovas, Abani Patra, Rebecca Batorsky

## Abstract

**Background:**

Population research increasingly relies on integrating data across studies to understand patterns of health and disease at broader scales. Such integration expands sample size and supports multimodal analyses, but independent cohort studies are often not directly comparable due to differences in instruments, timing, and measurement structure. Data harmonization addresses this challenge by transforming heterogeneous variables into comparable measures, allowing datasets collected under different protocols to be analyzed jointly, but published harmonization efforts seldom include an analysis conducted on the integrated dataset itself.

**Methods:**

We developed an integrated dataset, combining two longitudinal cohorts, the Boston Puerto Rican Health Study (BPRHS) and the Puerto Rico Observational Study of Psychosocial, Environmental, and Chronic Disease Trends (PROSPECT). Comparability between variables was established through an explicit framework distinguishing availability from construct comparability, and the evidence supporting comparability is reported by class of feature, including tests of measurement invariance and differential test functioning for the multi-item psychosocial scales. To support transparency and reuse, we provide an open-source Python pipeline and structured metadata describing variable alignment and transformations. As a worked example of downstream use, we conducted a Random Forest analysis predicting diabetes status.

**Results:**

The integrated dataset comprises 539 harmonized variables across four BPRHS visits and two PROSPECT visits, each documented with its source variable, question wording, and transformation. In the worked example, the integrated dataset retained predictive signal relative to cohort-specific models and improved performance for the smaller cohort.

**Conclusions:**

These findings illustrate how transparent, documented cross-cohort integration can support predictive population health research while making the basis for comparability explicit.

## Full Text Availability

The license terms selected by the author(s) for this preprint version do not permit archiving in PMC. The full text is available from the preprint server.

